# Economics of using beef semen on dairy herds

**DOI:** 10.3168/jdsc.2021-0155

**Published:** 2021-11-25

**Authors:** V.E. Cabrera

**Affiliations:** Animal and Dairy Sciences Department, University of Wisconsin-Madison, Madison 53706

## Abstract

The economic value of using beef semen in dairy herds depends on the market value of calves (crossbred beef and dairy), market price of semen (beef, conventional, and sexed), herd reproductive performance, and semen combination strategies. Due to the complex interaction among all these factors and their inherent changing conditions, the quest for an optimal strategy is best served by the application of an integrated model and a decision support tool adaptable to ever-changing farm and market conditions. We have developed a model and a decision support tool to calculate the income from calves over semen costs (ICOSC) in response to user-defined beef semen crossbreeding strategies in combination with sexed and conventional semen utilization. The model follows a Markov-chain approach in which animal (heifer and cow) statuses (age, months after calving, lactation, pregnancy, calving) are simulated monthly. Replacement balance is calculated as the difference between demand and supply of calves in function of selected semen utilization protocols, which could include beef, sexed, or conventional semen. A case study was performed in a 1,000-cow virtual Holstein herd with 35% turnover rate and 7% stillbirth rate. Five strategies of beef semen utilization on adult cows (0 to 100% in 25-percentage-unit intervals) were combined with 6 strategies of sexed semen use [none (NS), first service in heifers (1H), first and second services in heifers (2H), 2H + 20% top genetic cows (TOP), 2H + first service in primiparous (1C), and 1C + first service in second-lactation cows (2C)]. All animals not bred to either sexed or beef semen were bred to conventional semen. Having a price of beef calves ~4 times greater than the price of a dairy calf and having the price of sexed semen ~2.3 times greater than the conventional or beef semen determined that the optimal breeding semen protocols that concurrently maximized the ICOSC and produced enough replacements were 100% beef semen use after 2C sexed semen protocol (ICOSC = $2,001) for medium reproductive performance (~20% 21-d pregnancy rate) and 100% beef semen after 1H sexed semen protocol (ICOSC = $6,215) for high reproductive performance (~30% 21-d pregnancy rate). These strategies were consistently the best options under several feasible market conditions for herds with medium and high reproductive performance. However, the optimal ICOSC was negative or marginally low for low-performance herds (~15% 21-d pregnancy rate), for which the opportunity to use beef semen is minimal or nonexistent.

## Highlights

• Crossbred beef on dairy is valuable when reproductive performance is better than average.
• Crossbred beef on dairy is valuable when crossbred beef calves are more valuable than dairy calves.
• The value of beef semen is greater when used in combination with dairy sexed semen.
• The value of beef semen is greater when opportunity and willingness to buy and sell calves exists.
• The decision support tool is available online: https://DairyMGT.info: Tools: Premium Beef on Dairy Program.

## Graphical Abstract

**Figure:**
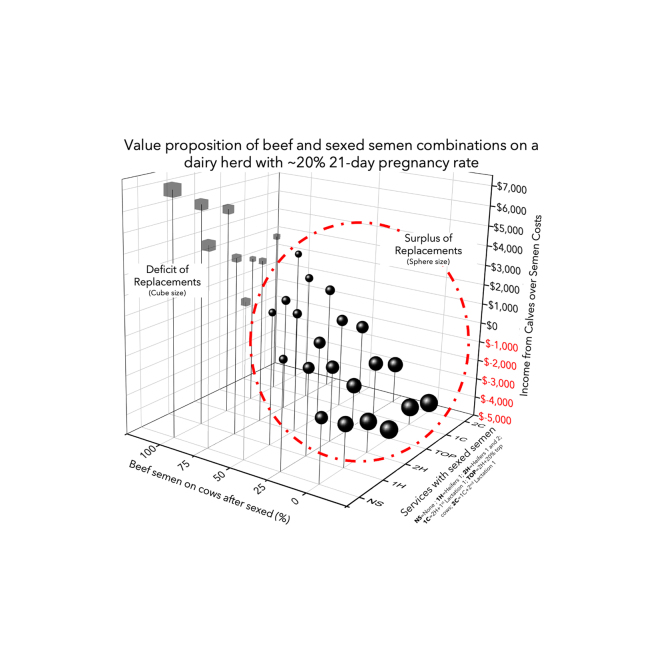
**Summary:** The difference between the extra revenue from selling crossbred beef (and dairy) calves and the extra costs incurred for beef (and sexed and conventional) semen can be defined as the income from calves over semen costs (ICOSC). An analysis of ICOSC under different market conditions indicated that the use of beef semen on dairy is an attractive business proposition for herds with at least a ~20% 21-d pregnancy rate when the price of crossbreed calves is at least double the average price of dairy calves, even when the sexed semen price is more than double the conventional and beef semen price.

Improved reproduction performance, increased use of female sex-sorted (sexed) semen, and production of more than enough dairy replacements together with attractive market value of crossbred beef calves have created favorable conditions to produce high-value crossbred beef calves using a selected group of dairy animals. Although the general economic proposition of using beef semen in dairy is appealing and beneficial (Pahmeyer and Britz, 2020) and it is growing in popularity among dairy farmers (Berry, 2021), no systematic economic study has quantified the economic value of using beef semen in dairy cows, nor has any study analyzed the interactions of farm reproductive efficiency and its opportunities for using beef semen. The economic value of using beef semen in dairy herds depends on the market value of calves (crossbred beef and dairy), market price of semen (beef, conventional, and sexed), herd reproductive performance, and semen combination strategies, all of which can be captured in the income from calves over semen costs (**ICOSC**). Due to the highly integrated nature of these factors and their ever-changing conditions, analyses warrant projection models, scenario evaluations, and sensitivity analyses. As such, the Premium Beef on Dairy model and decision support system tool from the University of Wisconsin-Madison (https://dairymgt.info/; Lopes and Cabrera, 2014) has been developed and updated on multiple occasions (Mur-Novales and Cabrera, 2017; Li and Cabrera, 2019b) with the aim of addressing questions related to the utilization of beef semen in dairy cattle. The main goal of this paper was to report a systematic analysis to determine the best (beef, sexed, and conventional) semen breeding strategies according to a herd's reproductive performance and market conditions using the aforementioned model and decision support system.

Following Cabrera (2012) and Li and Cabrera (2019b), a virtual herd of 1,000 Holstein cows was simulated through monthly Markov chains following the states of month of age (nulliparous) or month after calving and lactation (cows), and month in pregnancy using predefined monthly transition probabilities pertaining to culling risk that mimicked a herd with a 35% overall culling risk and 7% stillbirth rate (Li and Cabrera, 2019b). The other matrices of monthly transition probabilities pertaining to reproductive performance for heifers and cows were defined as part of the specific analyses. Animals became eligible to be bred at the beginning of mo 15 of age (heifers) or 3 mo after calving (cows). Reproductive events continued until the animal became pregnant or missed the opportunity to become pregnant (>22 mo of age in heifers and >10 mo after calving in cows). First, the Markov chain model was run until it reached steady state (Cabrera, 2012), assuming that all breedings were performed with conventional semen under a selected reproductive performance level. At that time, the monthly flow of dairy female calves required to maintain a constant adult herd size was calculated, as well as a matrix of the monthly numbers of breed-eligible heifers and cows by service (Table 1). Breed-eligible animals were used to evaluate dairy calf replacements produced and ICOSC by alternative semen utilization protocols under 3 different reproductive performance levels (low, medium, and high), assuming service rates of 75% for heifers and 60% for adult cows. The approximate 21-d pregnancy rate of cows was 30% for high, 20% for medium, and 15% for low reproductive performance.

**Table 1:** Breed-eligible animals (head/month), reproductive performance (conception rate in parentheses) of conventional semen, and flow of female replacement calves required to maintain constant the 1,000-cow herd size under high, medium, and low reproductive performance levels^1^

Animals	Service	Reproductive performance of breed-eligible animals, head/month (conception rate, %)		
		High	Medium	Low
Heifers	First	43 (60)	36 (55)	31 (50)
	Second	24 (55)	21 (50)	19 (45)
	Third	15 (50)	14 (45)	14 (40)
	> Third	29 (40)	31 (40)	34 (30)
Lactation 1	First	22 (45)	27 (40)	31 (35)
	Second	12 (40)	19 (35)	24 (30)
	Third	7 (35)	13 (30)	19 (25)
	> Third	16 (25)	35 (20)	55 (20)
Lactation 2	First	17 (40)	19 (35)	19 (30)
	Second	10 (35)	13 (30)	15 (25)
	Third	6 (30)	9 (25)	11 (20)
	> Third	12 (20)	23 (15)	32 (15)
Lactation > 2	First	29 (35)	24 (30)	19 (25)
	Second	16 (30)	16 (25)	15 (20)
	Third	9 (25)	11 (20)	11 (15)
	> Third	27 (15)	39 (15)	43 (10)
		Required calves per month		
Dairy female replacement calves		33	39	47

1 High (~30%), medium (~20%), and low (~15%) 21-d pregnancy rate; culling risk was 35% and stillbirth rate was 7%.

Fertility of beef semen was assumed to be the same as that of conventional semen (Mur-Novales and Cabrera, 2017), whereas fertility of dairy sexed semen was assumed to be 80% of that of conventional semen (Seidel, 2014). Percentages of female calves from conventional and sexed semen were set at 47% (Silva del Río et al., 2007) and 90% (Holden and Butler, 2018), respectively.

The ICOSC was calculated in US dollars per month ($/mo) as the sum product of all the calves produced times their values minus the sum product of all semen doses used times their prices. The ICOSC included a factor to add the value of sold or to subtract the value of purchased female replacement calves if they were in excess or deficit, respectively, according to the calculated female calf balance (calves produced minus calves required).

Baseline semen and calf prices (Lopes and Cabrera, 2014; Mur-Novales and Cabrera, 2017; Li and Cabrera, 2019b) were set at $15/semen dose of conventional or beef semen, $35/semen dose of Holstein sex-sorted dairy semen, $45/Holstein female calf, $57.5/Holstein male calf, and $225/crossbred beef calf.

Five beef semen use strategies were combined with 6 sexed semen use strategies. Beef semen utilization was restricted to eligible adult cows not being inseminated with sexed semen at percentages between 0 and 100% in 25% intervals: (1) 0%, (2) 25%, (3) 50%, (4) 75%, and (5) 100%. The sexed semen strategies included (1) no use of sexed semen (**NS**); (2) first service in heifers (**1H**); (3) first and second services in heifers (**2H**); (4) first and second services in heifers and 20% cows with top genetics at each service (**TOP**); (5) first and second services in heifers and first service in primiparous cows (**1C**); and (6) first and second services in heifers and first service in primiparous and second-parity cows (**2C**). Conventional semen was used on eligible animals that were not bred to either sexed or beef semen.

Thirty possible pairwise outcomes (ICOSC and replacement balances) resulted from each combination of beef and sexed semen strategy under a defined set of prices and for each level of reproductive performance (low, medium, and high). These outcomes for baseline market conditions are presented in Table 2.

**Table 2:** Income from calves over semen costs (ICOSC, $) and female calf replacement balance (produced minus required replacement calves, numbers in parentheses) for different strategies of beef and sexed semen use under different reproductive performance levels^1^

Reproductive performance and beef semen use^2^	Dairy sex-sorted semen use^3^					
	NS	1H	2H	TOP	1C	2C
Low performance						
0%	−3,246 (−4)	−4,094 (0)	−4,609 (2)	−5,936 (5)	−5,361 (5)	−5,814 (6)
25%	−1,008 (−10)	−1,856 (−6)	−2,372 (−4)	−4,146 (0)	−3,505 (0)	−4,160 (1)
50%	1,229 (−16)	381.3 (−12)	−134 (−10)	−2,356 (−5)	−1,649 (−5)	−2,507 (−3)
75%	3,467 (−22)	2,619 (−18)	2,103 (−16)	−565.9 (−10)	207 (−10)	−853 (−8)
100%	5,704 (−28)	4,856 (−24)	4,341 (−22)	1,224 (−14)	2,062 (−16)	801 (−12)
Medium performance						
0%	−1,790 (9)	−2,816 (14)	−3,403 (16)	−4,548 (19)	−4,068 (19)	−4,519 (20)
25%	**445**(**3**)	−581 (8)	−1,168 (10)	−2,760 (14)	−2,209 (13)	−2,889 (15)
50%	2,680 (−4)	**1,654**(**2**)	**1,067**(**4**)	−972 (9)	−350 (8)	−1,259 (11)
75%	4,915 (−10)	3,889 (−5)	3,302 (−2)	**816**(**4**)	**1,509**(**3**)	**371**(**7**)
100%	7,150 (−16)	6,124 (−11)	5,537 (−8)	2,605 (−1)	3,368 (−2)	**2,001**(**2**)
High performance						
0%	−503 (16)	−1,738 (23)	−2,400 (26)	−3,266 (28)	−2,952 (28)	−3,377 (29)
25%	**1,486**(**11**)	**250**(**17**)	−411 (20)	−1,676 (24)	−1,308 (23)	−1,973 (25)
50%	**3,474**(**5**)	**2,239**(**12**)	**1,577**(**15**)	−85 (20)	**336**(**19**)	−569 (22)
75%	**5,462**(**0**)	**4,227**(**6**)	**3,565**(**10**)	**1,506**(**15**)	**1,980**(**14**)	**835**(**18**)
100%	7,450 (−5)	**6,215**(**1**)	**5,553**(**4**)	**3,096**(**11**)	**3,624**(**10**)	**2,239**(**14**)

1 Bolded pairs of numbers indicate a combination in which both the ICOSC and the replacement balance (in parentheses) are positive.

2 High (~30%), medium (~20%), and low (~15%) 21-d pregnancy rate.

3 Dairy sexed semen use: NS = no use; 1H = first service in heifers; 2H = first and second services in heifers; TOP = first and second services in heifers and 20% cows with top genetics; 1C = first and second services in heifers and first service in primiparous cows; and 2C = first and second services in heifers and first service in primiparous and second-parity cows. Beef semen use only on adult cows and after sexed semen use. All remaining services to eligible animals that were not bred to beef or sexed semen were bred with conventional semen.

Greater use of beef semen from 0 to 100% increased the ICOSC regardless of sexed semen utilization and reproductive performance. In contrast, increased utilization of dairy sexed semen from NS to 2C reduced the ICOSC regardless of reproductive performance and use of beef semen. The highest ICOSC occurred with 100% beef semen and NS, whereas the lowest ICOSC occurred with 0% beef semen and 2C, regardless of reproductive performance.

Reproductive performance had a large effect on both ICOSC and calf balance. The better the reproductive performance, the more positive the ICOSC and the greater the replacement calf balance value. High reproductive performance determined the highest ICOSC for all breeding strategies, which is consistent with the relationship between profit and reproductive performance (Cabrera, 2014). For the low reproductive performance group, no breeding protocol achieved both positive ICOSC and replacement balance. However, out of the 30 sexed and beef semen use combinations, 7 combinations under medium reproductive performance and 17 combinations under high reproductive performance had both positive ICOSC and replacement balance (Table 2; bolded values). Certainly, beef semen utilization is limited by reproductive performance. It is clear that an inverse relationship exists between ICOSC and replacement balance, such that greater ICOSC are achieved when the replacement balance is lower, which also coincides with greater utilization of beef semen. It is also evident that greater beef semen use determines greater net return (Barrientos-Blanco et al., 2018) and stimulates more sexed semen use (Ettema et al., 2017; Bittante et al., 2020).

Because dairy sexed semen is expensive with respect to beef or conventional semen, increased utilization negatively affects the ICOSC. Also, because of high crossbred beef calf prices compared with Holstein female calf prices, more beef semen utilization increases the ICOSC while producing fewer female Holstein calves, which is consistent with previous reports (Mur-Novales and Cabrera, 2017; Li and Cabrera, 2019b).

Although under each reproductive performance, a positive ICOSC can be achieved, in many situations this is achieved only at the expense of buying extra replacements in the market (negative replacement balance). This happens because of the lower price of Holstein calves compared with crossbred beef calves. In other words, economically, it could make sense to produce high-value crossbred beef calves and purchase dairy calf replacements from outside. However, this might not be practical, realistic, or feasible for many reasons; for example, the lack of a consistent and reliable market for dairy replacement calves, biosecurity risks due to unknown origin of replacements, or genetic base loss due to unknown genetics of purchased replacements. Dairy farmers in Wisconsin (personal communication) voice their desire to take advantage of using beef semen while still producing enough on-farm female calf replacements. Farmers prefer to produce their own on-farm replacements, which counterbalances the economic opportunity of using beef semen (Li and Cabrera, 2019b). If farmers need to produce enough on-farm replacements, farmers will only select positive replacement balance strategies from Table 2.

Ideal situations—in which both the ICOSC and the replacement balance are positive—are bolded in Table 2. Out of these ideal situations, the optimal breeding protocol can be defined as that with the maximum ICOSC. As noted, due to the interaction of ICOSC and replacement balance, this optimal protocol happens when the minimum positive replacement balance occurs. Although there is no ideal or optimal situation for a low reproductive performance herd, the optimal breeding protocol for medium reproductive performance is when 100% beef semen is combined with 2C sexed semen, attaining an ICOSC value of $2,001 and 2 extra replacements. The optimal breeding protocol for high reproductive performance occurs when 100% beef semen is combined with 1H sexed semen, attaining an ICOSC value of $6,215 and 1 extra replacement (Table 2). These optimal breeding protocols are, in general, consistent with those found by Clasen et al. (2021), who reported the highest total economic return by using sexed semen in heifers and first-parity cows and beef semen on all other breedings. Different from the study presented here, Clasen et al. (2021) included the use of genomic testing to select calves out of the herd.

A practical evaluation to inform decisions according to a herd's reproductive performance under feasible but ever-changing market conditions can be performed following a sensitivity analysis of the best combination of beef and sexed semen combinations (best breeding protocol) to yield the maximum ICOSC while producing enough on-farm replacements (Table 3). As portrayed in Table 3, all optimal breeding strategies resorted, in all cases, to sexed semen use to allow more beef semen utilization and still produce enough replacements.

**Table 3:** Optimal beef and sexed semen protocol that maximizes the income from calves over semen costs (ICOSC, $) when it produces enough dairy female calf replacements under different market prices scenarios for herds differing in reproductive performance^1^

Performance^2^ and scenario	Semen cost, $/dose		Holstein calf, $/head		Beef calf, $/head	Best semen protocol^3^		Maximum ICOSC, $
	Sexed	Beef	Female	Male		Sexed	Beef, %	
Low performance								
Baseline prices	35	15	45	57.5	225	1H	0	−4,094
Wisconsin May 2021 price^1^	35	15	**28**	**110**	**275**	1H	0	−1,911
May 21 + high beef calf prices	35	15	**28**	**110**	**350**	TOP	25	−1,410
Conservative prices next 5 years	35	15	**38**	**90**	**250**	TOP	25	−2,992
Low beef semen price	35	**5**	45	57.5	225	2C	25	−3,552
Low beef and sexed semen price	**20**	**5**	**28**	**110**	**275**	TOP	25	40
Breakeven beef calf price	35	15	**28**	**110**	**487**	TOP	25	—
Medium performance								
Baseline prices	35	15	45	57.5	225	2C	100	2,001
Wisconsin May 2021 price^1^	35	15	**28**	**110**	**275**	2C	100	4,567
May 21 + baseline beef calf prices	35	15	**28**	**110**	225	1H	50	3,265
Conservative prices next 5 years	35	15	**38**	**90**	**250**	2C	100	3,373
Low beef semen price	35	**5**	45	57.5	225	2C	100	4,028
Low beef and sexed semen price	**20**	**5**	28	110	275	2C	100	8,147
Breakeven beef calf price	35	15	**28**	**110**	**100**	1H	50	—
High performance								
Baseline prices	35	15	45	57.5	225	1H	100	6,215
Wisconsin May 2021 price^1^	35	15	**28**	**110**	**275**	1H	100	9,493
May 21 + baseline beef calf prices	35	15	**28**	**110**	225	1H	100	7,199
Conservative prices next 5 years	35	15	**38**	**90**	**250**	1H	100	7,975
Low beef semen price	35	**5**	45	57.5	225	1H	100	8,688
Low beef and sexed semen price	**20**	**5**	28	110	275	1H	100	11,966
Breakeven beef calf price	35	15	**28**	**110**	**69**	1H	100	—

1 Bolded values indicate a change from the baseline prices scenario.

2 High (~30%), medium (~20%), and low (~15%) 21-d pregnancy rate.

3 Dairy sexed semen use: 1H = first service in heifers; TOP = first and second services in heifers and 20% cows with top genetics; and 2C = first and second services in heifers and first service in primiparous and second-parity cows. Beef semen use only on adult cows and after sexed semen use. All remaining services to eligible animals that were not bred to beef or to sexed semen were bred with conventional semen.

Low reproductive performance is a major hurdle to use beef semen and produce high-value crossbred beef calves. In these herds, the optimal ICOSC was negative under current and most projected market conditions. These herds struggle to produce enough replacements even without using beef semen. When forced to produce enough replacements under baseline or May 2021 market conditions, they were required to use sexed semen on the first breeding of heifers (1H) with no use of beef semen, resulting in negative ICOSC. Even with favorable market conditions of a high price of $350/crossbreed calf, low reproductive herds would still have a negative ICOSC, in which the best protocol is 25% beef semen with TOP sexed semen use. The only scenario in which low reproductive performance herds would have a positive ICOSC is when Holstein male and crossbred beef calf prices are high and sexed and beef semen prices are extremely low, which is unlikely. A breakeven analysis for these low reproductive performance herds demonstrates that, under other May 2021 market conditions, they would require a market price of crossbred beef calves to be at least $487/head for beef/sexed semen use to become an economic option. Such a high price for a crossbred beef calf is unrealistic related to historical and projected prices (Li and Cabrera, 2019a). In short, low reproductive performance herds have no economic opportunity to produce crossbred beef calves. These herds would first need to improve their reproductive performance.

The figure is substantially different for medium reproductive performance herds, for which all estimated ICOSC are positive. These herds would be required to use between 50 and 100% beef semen with 1H to 2C sexed semen. The ICOSC with baseline market conditions yields $2,001, a value that increases to $3,373 under conservative projected market conditions. The breakeven crossbred beef calf price is only $100/head, a price much lower than historical records and projected prices (Li and Cabrera, 2019a). The opportunity of using beef semen on medium reproductive herds is large and will remain a good business opportunity for the foreseeable future if price projections from the Food and Agricultural Policy Research Institute (https://www.fapri.missouri.edu) hold (Li and Cabrera, 2019a).

The situation is even more encouraging for high reproductive performance herds, in which the ICOSC is highly positive under almost any feasible market situation and would require that the price of the crossbred beef calf be only $69/head to break even, similar to the current average price of male and female Holstein calves. In these herds, because they already produce more than enough replacements, the use of beef semen could be justified even without any use of sexed semen. However, a better economic return is obtained when sexed semen is used on the first heifer breedings (1H). In all cases, the best optimal semen protocol is to use 100% beef semen in the adult herd.

It is important to note that the volatility of prices, especially that of calf prices (Lord et al., 2015), and the price ratio between beef crossbred and Holstein calves are key drivers for final outcomes and decisions. Thus, as market prices and herd performance change, recurrent use of a decision support tool such as the Premium on Beef Dairy becomes even more relevant. The decision support tool resulting from the model used in the current paper is openly and freely available at the Dairy Management website from the University of Wisconsin-Madison (https://DairyMGT.info: Tools: Premium Beef on Dairy). It can be used to evaluate ICOSC and heifer replacement balance for particular herd reproductive and management characteristics under specific market conditions.

It must be emphasized that this study did not include the effect of genetic gain, gestation length, or calving difficulty (dystocia) due to beef semen use on dairy cattle. These factors could have an economic impact on the use of beef semen on dairy. Use of beef (and sexed) semen could increase selection intensity and reduce the interbreeding interval, both of which promote faster genetic progress (Berry, 2021) and therefore increase the economic value of using beef semen on dairy in the long term. The analyses presented in this paper are intended for decisions in the short to mid term (9 to 12 mo), a period for which genetic progress can be neglected. Regarding gestation length, Scanavez and Mendonça (2018) found that the Holstein gestation period was slightly longer for cows that conceived from Angus beef sires (276.5 ± 0.6 d) than for cows that conceived from Holstein sires (274.9 ± 0.6 d). However, the difference was only 1.6 d, which can be disregarded for practical decision-making purposes. Concerning dystocia, the incidence of calving difficulty is slightly greater when using beef semen on dairy (Bittante et al., 2020); however, it seems that its effect is not large enough to change the major decisions (Clasen et al., 2021). Overall, it is safe to say that the impact of genetic gain, gestation length, and calving difficulty are small enough that they would not change the direction of the results or the main take-home messages in the short and mid term. Nonetheless, model improvements are underway to accommodate long-term assessments and the inclusion of genetic progress, use of genomic tests on calves, and other performance and health implications during pregnancy and during subsequent lactations.
